# Canine Adipose-Derived Mesenchymal Stem Cells (cAdMSCs) as a “Trojan Horse” in Vaccinia Virus Mediated Oncolytic Therapy against Canine Soft Tissue Sarcomas

**DOI:** 10.3390/v12070750

**Published:** 2020-07-12

**Authors:** Ivan Petrov, Ivaylo Gentschev, Anna Vyalkova, Mohamed I. Elashry, Michele C. Klymiuk, Stefan Arnhold, Aladar A. Szalay

**Affiliations:** 1 Department of Biochemistry/Cancer Therapy Research Center (CTRC), Theodor-Boveri-Institute, University of Wuerzburg, 97074 Wuerzburg, Germany; anna.vyalkova@uni-wuerzburg.de; 2 Institute of Veterinary-Anatomy, -Histology and -Embryology, Faculty of Veterinary Medicine, Justus-Liebig-University Giessen, 35392 Giessen, Germany; Mohammed.Elashry@vetmed.uni-giessen.de (M.I.E.); Michele.Klymiuk@vetmed.uni-giessen.de (M.C.K.); 3 Department of Radiation Oncology, Rebecca & John Moores Comprehensive Cancer Center, University of California, San Diego, La Jolla, CA 92093, USA

**Keywords:** oncolytic virus, cancer, vaccinia virus, canine cancer cell lines, canine adipose-derived mesenchymal stem cells (cAdMSCs), canine soft tissue sarcoma (CSTS), canine cancer therapy

## Abstract

Several oncolytic viruses (OVs) including various human and canine adenoviruses, canine distemper virus, herpes-simplex virus, reovirus, and members of the poxvirus family, such as vaccinia virus and myxoma virus, have been successfully tested for canine cancer therapy in preclinical and clinical settings. The success of the cancer virotherapy is dependent on the ability of oncolytic viruses to overcome the attacks of the host immune system, to preferentially infect and lyse cancer cells, and to initiate tumor-specific immunity. To date, several different strategies have been developed to overcome the antiviral host defense barriers. In our study, we used canine adipose-derived mesenchymal stem cells (cAdMSCs) as a “Trojan horse” for the delivery of oncolytic vaccinia virus Copenhagen strain to achieve maximum oncolysis against canine soft tissue sarcoma (CSTS) tumors. A single systemic administration of vaccinia virus-loaded cAdMSCs was found to be safe and led to the significant reduction and substantial inhibition of tumor growth in a CSTS xenograft mouse model. This is the first example that vaccinia virus-loaded cAdMSCs could serve as a therapeutic agent against CSTS tumors.

## 1. Introduction

The use of oncolytic virus strains is a novel approach for the treatment of canine cancer patients. Several different oncolytic viruses (OVs) have successfully been tested in the treatment of canine tumors [1,2,3,4]. One of the most important criteria for successful cancer therapy is the ability of the oncolytic virus to overcome different components of the host immune system including neutralizing antibodies and complementing adaptive antiviral immune cells. In order to evade virus inactivation by these factors, several strategies have been investigated, one of which includes the use of carrier systems for delivery of the oncolytic viruses. Different types of cells, such as immune cells [5], stem cells [6,7,8], and tumor cells [9], have successfully been utilized as carriers to deliver OVs to tumors. In addition, virus coatings with biocompatible polymers such as silk-elastin-like protein [10], polyethylene glycol (PEG) [11], and serum proteins [12,13], showed minimal sequestration by the mononuclear phagocytic system in the liver and spleen.

In general, carrier-mediated deliveries of OVs may protect the virus against the components of the host innate or acquired immune system and may increase the efficacy of viral tumor colonization. In our study, we examined canine adipose-derived mesenchymal stem cells (cAdMSCs) as a carrier for delivery of oncolytic vaccinia virus strains. These cells are relatively easy to isolate from canine adipose tissue and have similar immunomodulatory potency compared to canine bone marrow-derived mesenchymal stem cells [14,15]. In addition, the innate tropism of the mesenchymal stem cells for tumors makes these cells particularly effective for the cellular delivery of anti-cancer drugs [16,17]. Most importantly, Draganov and colleges recently demonstrated that AdMSCs protect the vaccinia virus from both innate and adaptive antiviral immunity [18].

Our data showed that vaccinia virus-loaded cAdMSCs have abilities to home to sites of tumor growth and to substantially inhibit the tumor growth in canine soft tissue sarcoma xenograft mouse model.

## 2. Materials and Methods

### 2.1. Ethics Statement

All mouse animal experiments were carried out in accordance with protocol approved by the government of Unterfranken, Germany, according to the German Animal Welfare Act (TierSchG) (permit numbers: RUF-55.2-DMS-2532-2-610). The canine soft tissue sarcoma (STSA-1) cell line was obtained from Dr. A. MacNeill (University of Colorado, USA) and was derived from a canine patient with a low-grade II soft-tissue sarcoma [19]. The prostate adenocarcinoma (CT1258) cell line was provided by Dr. I. Nolte (University of Veterinary Medicine, Hannover, Germany) [20]. Canine AdMSCs were obtained from adipose tissue from healthy canine donors and were described in this study. The isolation of cAdMSCs from adipose tissue was approved by the local authorities (approval no. V 54–19 c 20 15 h 02 GI 18/1 kTV 1/2018).

### 2.2. Viruses, Cells, and Cell Lines

Sucrose gradient purified C1-opt1, W1-opt1 or L3-opt1 (thymidine kinase (TK)-inserted Turbo-FP635 engineered Copenhagen (C1), Wyeth (W1) or LIVP (L3) strains) vaccinia viruses were provided by Tanja Auth, StemVac GmbH, Bernried, Germany.

Cells were cultured in Dulbecco’s Modified Eagle’s Medium (DMEM) supplemented with antibiotic-solution (100 U/mL penicillin G, 100 units/mL streptomycin) and 10% fetal bovine serum (FBS; GE Healthcare/PAA, Pasching, Austria) for African green monkey kidney fibroblasts (CV-1) cell line (ATCC, CCL-70) and 20% FBS for canine prostate carcinoma cell line CT1258 at 37 °C under 5% CO_2_.

STSA-1 cells were cultivated in minimum essential medium (MEM) with Earle’s salts supplemented with 2 mM of glutamine, 50 U/mL of penicillin G, 50 µg/mL of streptomycin, 1 mM of sodium pyruvate, 0.1 mM of nonessential amino acids (MEM-C), and 10% FBS.

The virus-infected cells were examined using an Axiovert 200M microscope (Carl Zeiss Microscopy GmbH, Gottingen, Germany) with Axiovision 4.5 software (138,861,040 pixel gray scale images). Digital images were processed with Photoshop 7.0 (Adobe Systems, Mountain View, CA, USA).

### 2.3. Adipose-Derived Stem Cells Isolation and Culture

Adipose tissues from dogs were obtained by surgical intervention and were isolated and cultured as described before [15]. In brief, adipose tissue was digested with Collagenase I (Biochrom, Berlin, Germany), filtered through a sterile 70-µm cell strainer (Greiner Bio-One, Frickenhausen, Germany), and cultured in DMEM low glucose (Fisher Scientific, Schwerte, Germany), supplemented with 10% fetal calf serum (LOT No. CP17-1688, Capricorn, Ebsdorfergrund, Germany) and 1% penicillin/streptomycin (Capricorn, Ebsdorfergrund, Germany). Nonadherent cells were discarded through subsequent culture passages. Finally, we obtained a homogenous cAdMSC culture.

After isolation and culture, cAdMSCs were characterized by trilineage differentiation as described elsewhere [21]. FACS analysis (BD Accuri C6) was performed from passage 3 cAdMSCs for stemness markers CD44 and CD90 and non-stemness markers CD45 and MHCII (Table 1), and results were evaluated with BD Accuri C6 software (Version 1.0.264.21). Dead cells were excluded from analysis after staining with 7-AAD (Cat. No. 559925, BD Biosciences, Heidelberg, Germany).

### 2.4. Viral Replication

For the viral replication assay, STSA-1, CT1258, or cAdMSC cells were infected with C1-opt1, W1-opt1, or L3-opt1 vaccinia virus strains at a MOI of 0.5. After one hour of incubation at 37 °C, the infection medium was removed and replaced by fresh growth medium. After 3 h, 24 h, 48 h, 72 h, and 96 h, the cells and supernatants were harvested. Following three freeze-thaw cycles and three times sonication (three times at 30 s), serial dilutions of the supernatants and lysates were tittered by standard plaque assay on CV-1 cells. All samples were measured in triplicate.

### 2.5. Lactate Dehydrogenase (LDH) Assay

The viral cytotoxicity in cAdMSCs was quantitatively assessed by LDH viability (Pierce LDH cytotoxicity Assay kit, Thermo Scientific, Dreieich, Germany) which is based on detecting lactate dehydrogenase released from damaged cells. For this purpose, cAdMSCs seeded in 24–well plates were infected with C1-opt1, W1-opt1, or L3-opt1 vaccinia virus strains at a MOI of 0.5. After one hour of incubation at 37 °C, the infection medium was removed and replaced by fresh growth medium. After 3 h, 24 h, 48 h, 72 h, and 96 h, cell-free supernatant aliquots were collected from each experimental sample and LDH in the culture supernatants was measured using the Pierce LDH Cytotoxicity Assay Kit, following the manufacturer’s instructions. These measurements were performed with Tecan Sunrise Remote microplate reader (Tecan, Maennedorf, Switzerland) at wavelengths of 490 nm and 680 nm. The results were shown as fractions of LDH release. Data were presented as mean standard deviation (SD), *n* = 3.

### 2.6. Western Blot Analysis

For detection of the virus-encoded proteins, STSA-1 or CT1258 cells were harvested and resuspended in SDS sample buffer at 24 h, 48 h, 72 h, or 96 h post virus infection (hpvi). Samples were separated by 10% SDS-Polyacrylamide gel electrophoresis and subsequently transferred onto a nitrocellulose membrane (Whatman GmbH, Dassel, Germany). After blocking in 5% skim milk in PBS, the membrane was incubated with rabbit polyclonal antibody against red turboFP635 protein (Cat. No. AB 231, Evrogen, Moscow, Russia) or polyclonal rabbit anti-vaccinia virus antibody (ab35219 Abcam, Cambridge, UK). The primary antibodies were detected using horseradish peroxidase-conjugated anti-rabbit (ab6721, Abcam, Cambridge, UK) secondary antibody, followed by enhanced chemiluminescence detection.

### 2.7. Animal Experiments

The STSA-1 xenograft tumors were generated by implanting 4 × 10^6^ canine soft tissue sarcoma (STSA-1) cells subcutaneously into the right hind leg of six- to eight-week-old female nude mice (Athymic Nude-Crl:NU(NCr)-Foxn1nu, Charles River, Sulzfeld, Germany). Tumor growth was monitored at least once a week in two dimensions using a digital caliper. Tumor volume was calculated as [(length × width^2^)/2]. When tumors reached 300–500 mm^3^, STSA-1 mice were randomized, distributed into three groups (*n* = at least 3), and injected with a single intravenous dose of either C1-opt1 alone (2.5 × 10^6^ pfu), cAdMSCs alone (2.5 × 10^5^ cells), or a combination of C1-opt1/cAdMSCs (2.5 × 10^6^ pfu C1-opt1/ 2.5 × 10^5^ cAdMSCs; MOI of 10), previously co-incubated for 2 h at 37 °C. Mice were monitored for changes in body weight and signs of toxicity. The significance of the results was calculated by two-way ANOVA at 0.05 level of significance. Results are displayed as means ± SD.

### 2.8. Histological Analysis of Tumors

For histological studies of the xenograft model, tumors were excised and snap-frozen in liquid nitrogen. Tissue samples were sectioned (7-mm thickness) with the cryostat CM3050 S (Leica Microsystems GmbH, Wetzlar, Germany). The VACVs were labeled using CODEX tag-conjugated polyclonal rabbit anti-vaccinia virus (anti-VACV) antibody (ab35219 Abcam, Cambridge, UK) and ATTO 550 tag-conjugated fluorescent dye (kindly provided by G. Nolan, Stanford, CA, USA). The fluorescence-labeled preparations were examined using a BZ-X800 fluorescence microscope (Keyence, Osaka, Japan) equipped with the BZ-X800 Analyzer software (Keyence, Osaka, Japan).

### 2.9. PCR Detection of Vaccinia Virus in Serum of Virus-Injected Mice

The presence of the C1-opt1 in the sera of virus-injected mice was determined by PCR analysis with specific primers for turboFP635 (Forward 5’ATGGTGGGTGAGGATAGCGTGCT3′ and Reverse 5′TCAGCTGTGCCCCAGTTTGCTAGG3′) and for hemagglutinin gene A56R (Forward 5′-ACGGCCGACAATATAATTAATGC-3′ and Reverse 5′-CATCATCTGGAATTGTCACTACTAAA-3). Sucrose gradient-purified C1-opt1 virus (Copenhagen strain) was used as PCR-positive control (detection LIMIT < 100 pfu/50 µL serum).

## 3. Results

### 3.1. Analysis of the Oncolytic Potential of C1-opt1, W1-opt1, and L3-opt1 Vaccinia Virus Strains against Canine Cancer Cells

**Figure 1:**
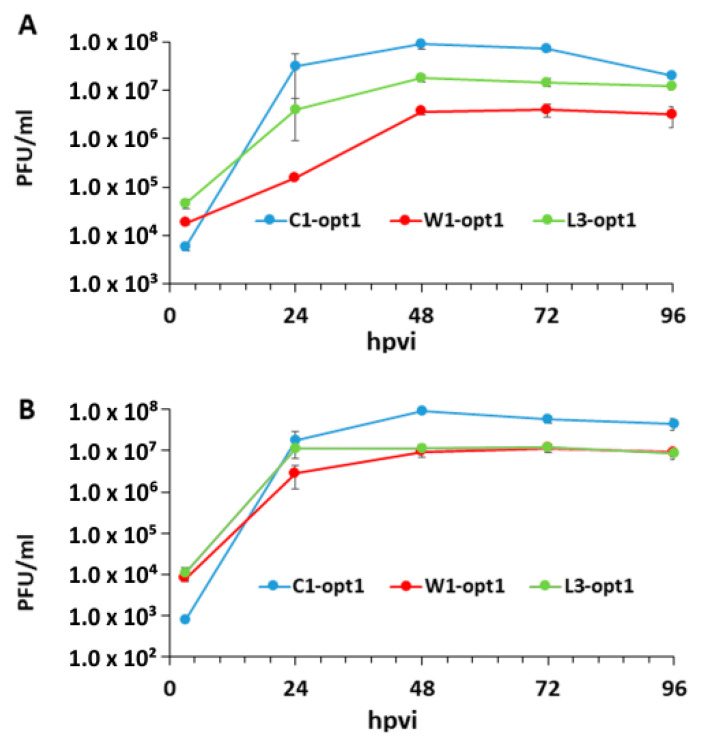
Replication efficiency of vaccinia virus strains, C1-opt1, W1-opt1, and L3-opt1, in canine cancer CT1258 cells (**A**) or STSA-1 (**B**) cells at a MOI of 0.5 at different timepoints.

The three new recombinant oncolytic vaccinia virus (VACV) strains used in this study, expressing the red turboFP635 fluorescent protein (FP635), were kindly provided by StemVAC GmbH, Bernried, Germany. The construction details of these strains will be published elsewhere. The oncolytic effects of VACVs were studied in a panel of two different canine cancer cell lines, including soft tissue sarcoma (STSA-1) [19] and prostate carcinoma (CT1258) [20]. Several experimental settings demonstrated that all tested VACV strains efficiently infected and replicated (>100-fold virus titer increase at 24 hpvi to 96 hpvi) in both canine cancer lines under cell culture conditions (Figure 1). The highest virus titer was identified in C1-opt1 virus infected CT1258 cells at 48 h post infection (9.18 × 10^7^ pfu/mL) (Figure 1B).

**Figure 2:**
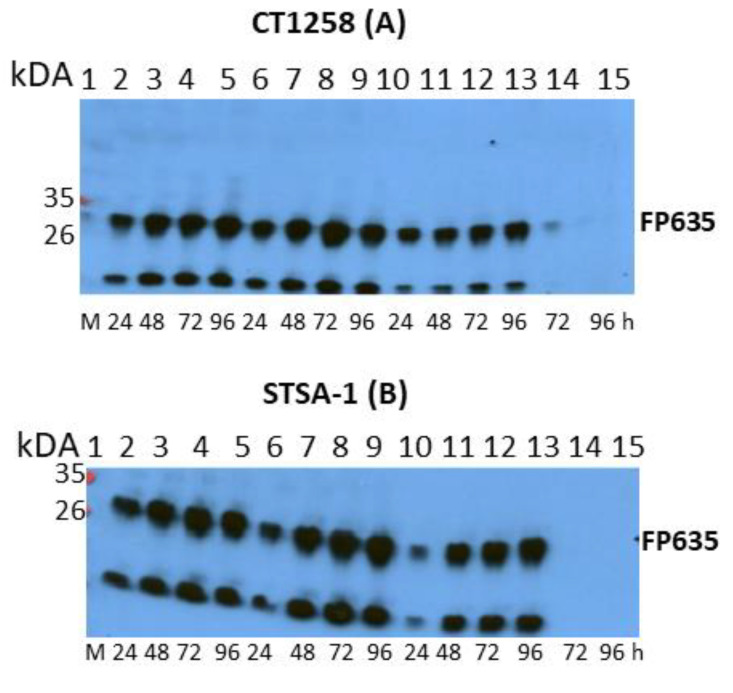
Western blot (WB) analysis of the time-dependent expression of virus mediated FP635 in vaccinia virus infected canine cancer CT1258 cells (**A**) or in canine soft tissue sarcoma STSA-1 cells (**B**). Lane M: Protein markers; lanes 2–5: L3-opt1-infected cells; lanes 6–9: C1-opt1-infected cells; lanes 10–13: W1-opt1-infected cells; lanes 14–15: noninfected CT1258 or STSA-1 cells (negative controls). Semi-quantitative WB analysis can be also found in Supplementary Figure S1. Note: The second protein band on the bottom of the blots was possibly a degradation FP635 product.

In order to confirm the virus replication in canine cancer cell lines CT1258 and STSA-1, we followed virus-mediated expression of FP635 by Western blot analysis (Figure 2 and Supplementary Figure S1). In these studies, we found that infection with C1-opt1 at a MOI of 0.5 exhibited the strongest expression of FP635 in both CT1258 (Figure 2A) and STSA-1 cells (Figure 2B).

### 3.2. Canine AdMSCs Provide Potent Amplification of Vaccinia Virus

**Figure 3:**
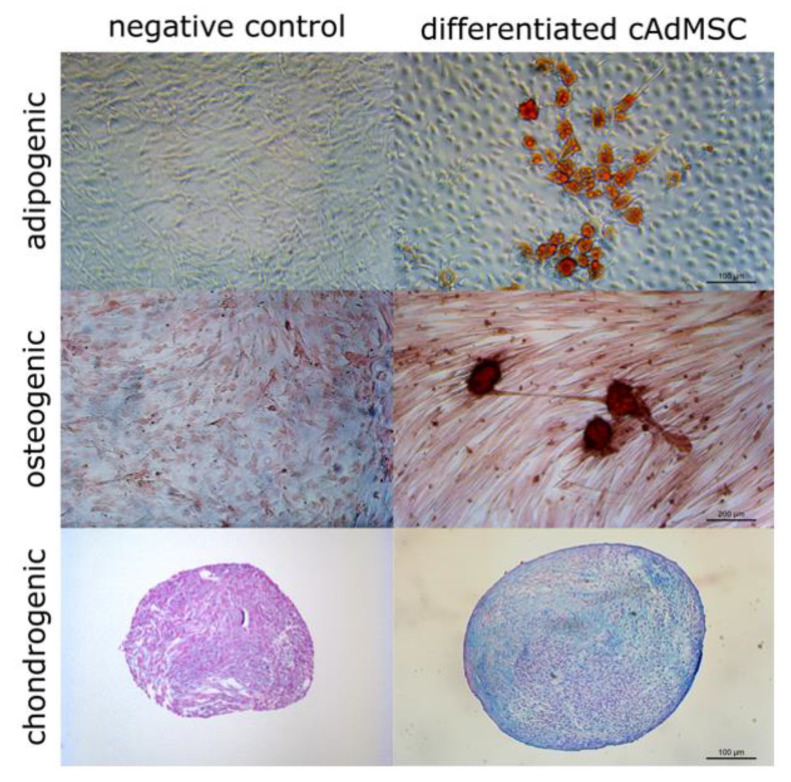
Differentiation of canine adipose-derived mesenchymal stem cells (cAdMSCs) in adipogenic, osteogenic, and chondrogenic lineages. Adipogenic and osteogenic differentiation are shown as monolayer in cell culture flasks, chondrogenic differentiation is shown as histological sections. Stained with Oil Red O (adipogenic), Alizarin Red S (osteogenic), or Alcian Blue (chondrogenic).

**Figure 4:**
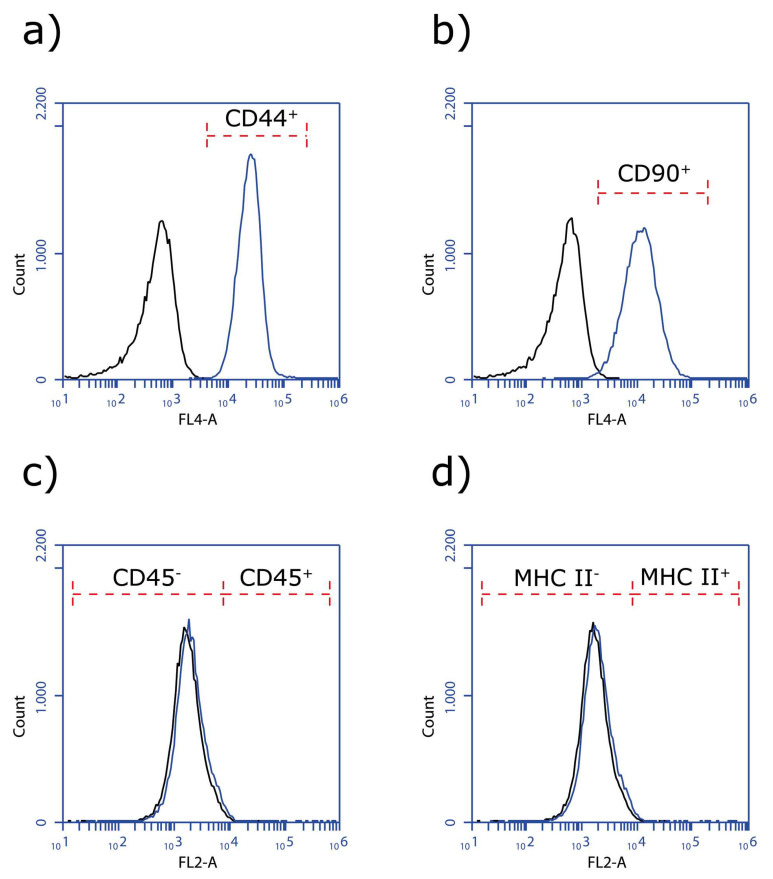
FACS analysis of stemness markers CD 44 (**a**), CD90 (**b**), and non-stemness markers CD45 (**c**), and MHC II (**d**) from cAdMSCs. Black line = corresponding isotype control, blue line = non-/stemness marker labeled cell counts.

Canine AdMSCs were obtained from the adipose tissue of healthy donors as described in the Material and Methods section. The cAdMSCs were characterized for their capability to differentiate into three main mesodermal lineages. This could be shown by formed lipid droplets (adipogenic), calcium deposits (osteogenic) and build glycosaminoglycans (chondrogenic) (Figure 3), and the expression profile of MSC markers determined via flow cytometry (Figure 4).

FACS analysis was performed from passage 3 (p3) of cAdMSCs with antibodies for stemness markers CD44, CD90, and non-stemness markers CD45 and MHCII (Table 1). CD44 and CD90 were expressed by 99% of the cAdMSCs, whereas virtually no expression of CD45 and MHC II was detected (Figure 4).

**Figure 5:**
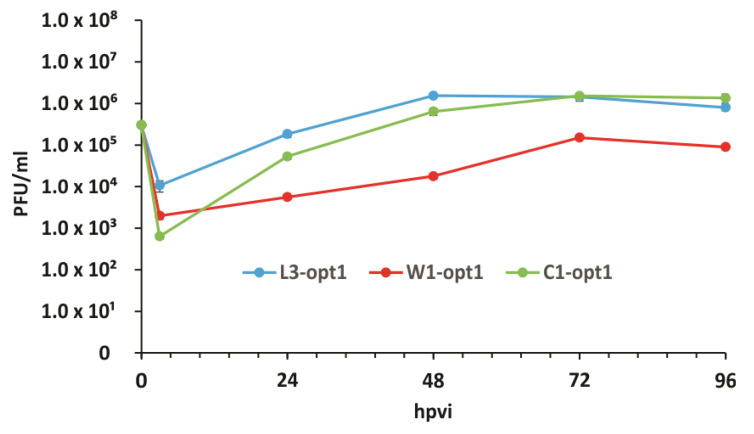
Replication efficiency of vaccinia virus strains, C1-opt1, W1-opt1, and L3-opt1, in cAdMSCs at a MOI of 0.5 at different timepoints.

In order to test the efficiency of vaccinia virus replication, p3-generated cAdMSCs cells were infected with C1-opt1, W1-opt1, and L3-opt1 viruses at a MOI of 0.5. Standard plaque assays were performed for all samples to determine the viral titers at different timepoints during the course of infection (Figure 5). The maximum viral titers were determined for L3-opt1 at 48 hpvi (1.52 × 10^6^ pfu/mL, five-fold increase compared to loaded virus at 0 hpi) and for C1-opt1 at 72 hpvi (1.51 × 10^6^ pfu/mL, five-fold increase compared to loaded virus at 0 hpi) (Figure 5).

**Figure 6:**
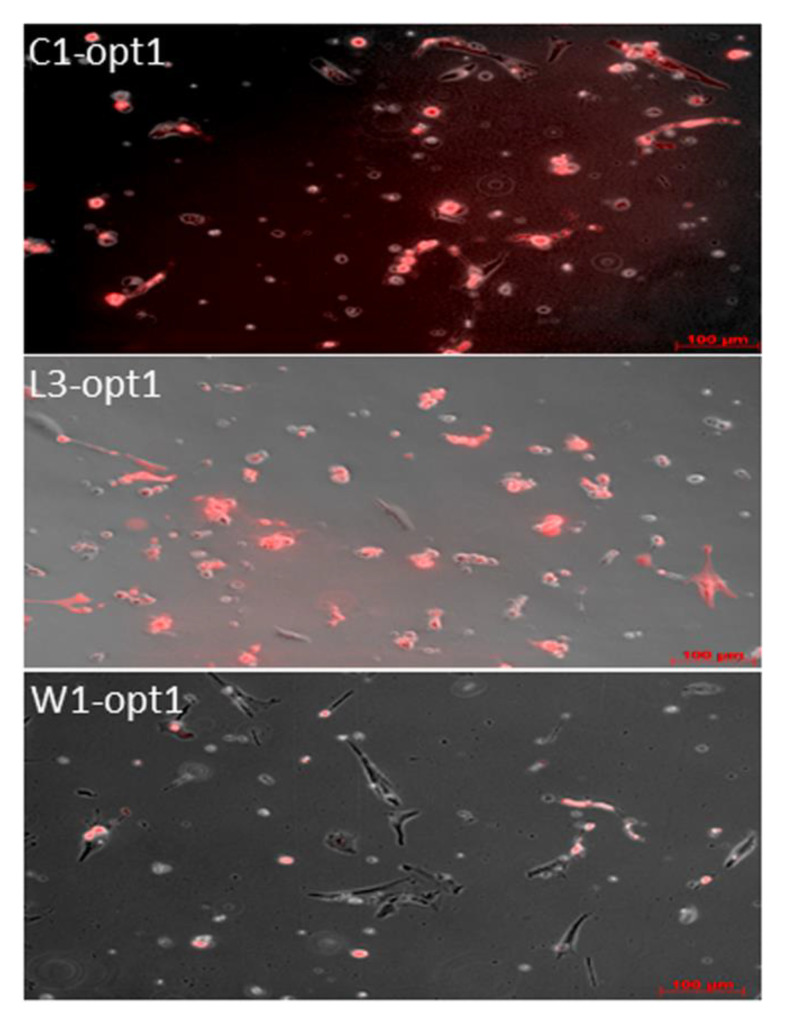
Fluorescence imaging of vaccinia virus infected cAdMSCs at 96 hpvi. Canine AdMSCs were infected with C1-opt1, W1-opt1, and L3-opt1 at MOI of 0.5. The virus infections were followed by monitoring of FP635 virus-mediated expression via fluorescence microscopy. All pictures in this set were taken at the same magnification. Scale bars represent 100 µm.

In these experimental settings, all viruses efficiently infected cAdMSCs, as demonstrated by the virus-mediated expression of the turboFP635 protein determined by fluorescence microscopy at 96 hpvi (Figure 6).

**Figure 7:**
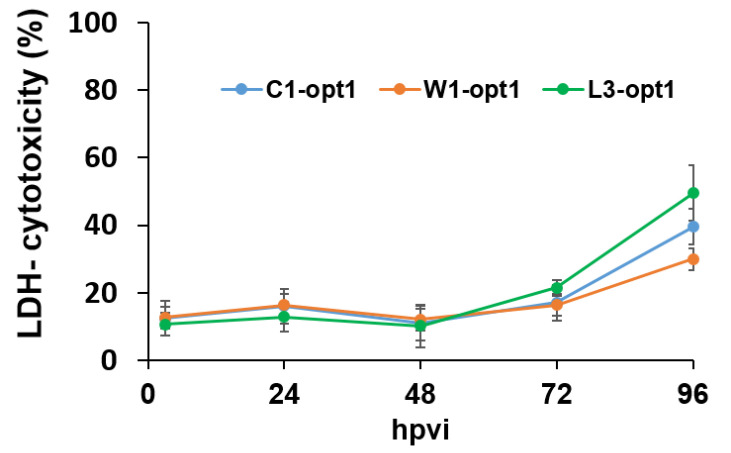
Time-dependent lysis of cAdMSCs after virus infection at a MOI of 0.5. The percentages of lysed cAdMSCs after infections with C1-opt1, W1-opt1, and L3-opt1 viruses were determined and calculated using the Pierce LDH cytotoxicity assay kit, following the manufacturer’s instructions. Mean values (*n* = 3) are shown as percentages of respective controls.

Finally, we also tested the cytolytic effects of the three viruses on the canine stem cells after infection using the Pierce LDH cytotoxicity assay kit (Thermo scientific, Dreieich, Germany). Surprisingly, we found that under these experimental conditions (MOI of 0.5) only about 30% to 50% of the infected stem cells were lysed at 96 hpvi (Figure 7).

In summary, our studies demonstrated that the used vaccinia virus strains infected und replicated in canine AdMSCs without a significant lysis (less than 15%) of the stem cells at least in the first two days of infection. Therefore, cAdMSCs could be useful as a “Trojan horse” for the delivery of VACVs. For animal experiments, we chose C1-opt1 (Copenhagen strain), since the strain was optimal concerning tumor cell lysis and replication in cAdMSCs.

### 3.3. A Single Systemic Application of C1-opt1 Vaccinia Virus Alone or in Combination with cAdMSCs Causes Significant Inhibition of Tumor Growth in STSA-1 Xenografts

The STSA-1 xenograft tumors were generated by implanting 4 × 10^6^ canine soft tissue sarcoma (STSA-1) cells subcutaneously into the right hind leg of seven-week-old female nude mice (Athymic Nude-Crl:NU(NCr)-Foxn1nu, Charles River Germany). Four weeks post implantation, all mice developed tumors with volumes of 200 mm^3^ to 500 mm^3^. Animals were separated into three groups (*n* = at least 3) and injected intravenously (i.v.) with a single dose of either C1-opt1, cAdMSCs, or a combination of both into the lateral tail vein. The cAdMSCs-injected mice were used as a control.

**Figure 8:**
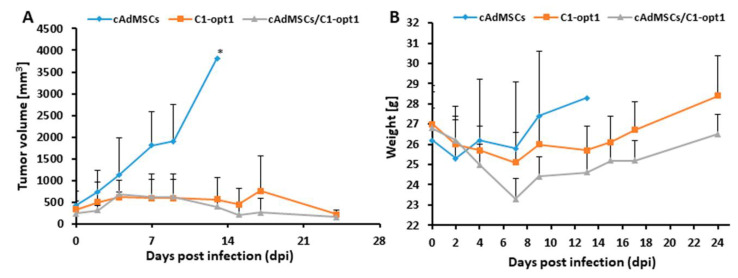
Effects of systemic C1-opt1 virus injection on tumor growth (**A**) and the body weights (**B**) of STSA-1 xenografted mice. The significance of the results was calculated by two-way ANOVA. Results are displayed as means ± standard deviation (SD), *p* values < 0.05 were considered significant. * indicates *p* = 0.05.

As shown in Figure 8, the virus treatment led to significant differences in tumor growth between cAdMSCs control and all virus-treated mice. Due to excessive tumor burden (>3000 mm^3^), all animals in the control cAdMSCs group were euthanized after 13 days post injection (dpi). At this timepoint, there was no significant difference between the two virus-treated groups (C1-opt1 vs. cAdMSCs/C1-opt1).

**Figure 9:**
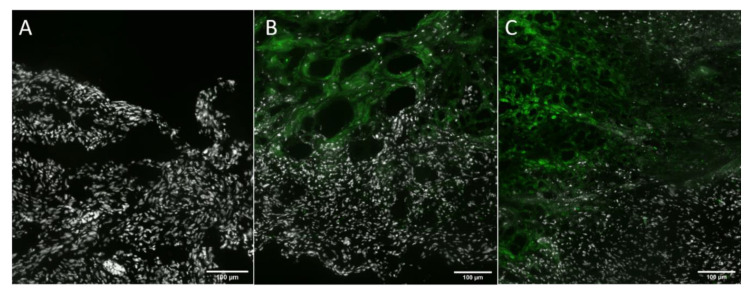
Visualization of viral tumor colonization by immunofluorescence-based imaging in tissue sections of primary tumors of STSA-1 tumor bearing mice injected with: (**A**) cAdMSCs control, (**B**) C1-opt1, and (**C**) cAdMSCs /C1-opt1. DAPI nuclear staining is indicated in white and the staining of vaccina virus is shown in green color. All pictures in this set were taken at the same magnification. Scale bars represent 100 µm.

The toxicity of the combination therapy was determined by monitoring the weight change of mice over time (Figure 9B). All virus- and/or cAdMSCs-treated mice showed stable mean weight over the course of studies. There were no signs of virus or cAdMSCs-mediated toxicity.

### 3.4. Virus Distribution in STSA-1 Tumor-Bearing Nude Mice after Virus Injection

In order to analyze the oncolytic effects of C1-opt1 virus alone and in combination with cAdMSCs in STSA-1 xenografts, we compared the virus colonization in different organs and tumor tissues. Table 2 summarizes the virus distribution data in sarcoma tumor-bearing nude mice at 24 dpvi. The highest viral titers were identified in primary tumors of cAdMSCs/C1-opt1-treated mice (Table 2). In the tumor tissues, there was no significant difference of the virus titers between C1-opt1 and cAdMSCs/C1-opt1-injected groups. Interestingly, we also found viral particles in lungs of most mice injected with cAdMSCs/C1-opt1 but not with C1-opt1 alone (Table 2). However, the detected C1-opt1 PFUs in tumors were about 10^3^–10^5^-fold higher when compared to PFUs in the lungs of corresponding animals at the same timepoint (Table 2). Taken together, our data clearly demonstrates that both C1-opt1 alone and the cAdMSCs/C1-opt1 combination display tumor specific replication in STSA-1 xenograft mice.

In addition, the presence of virus was analyzed by immunohistochemistry. For this purpose, tissue sections of primary tumors of STSA-1 tumor-bearing mice injected with C1-opt1, cAdMSCs/C1-opt1 at 24 dpvi and with cAdMSCs alone at 13 dpi were prepared as described in the Materials and Methods section. The histological data revealed a massive viral distribution in the tumor tissues of the C1-opt1- and C1-opt1/cAdMSCs-infected groups at the last stage of infection (Figure 9B,C). In both cases, the virus injection led to similar intratumoral virus replication and destruction of infected tumor cells, as shown by DAPI (4′, 6-diamidino-2-phenylindole) staining (Figure 9B,C). In contrast, no destruction of tumor cells was observed in the solely cAdMSCs-treated mice (Figure 9A). In summary, the results suggest that both C1-opt1 and cAdMSCs/C1-opt1 treatments led to a significant reduction and substantial inhibition of tumor growth in STSA-1 xenografts.

## 4. Discussion

In the current study, we investigated the oncolytic efficacy of three new VACVs, namely C1-opt1, W1-opt1, and L3-opt1, expressing the red turboFP635 protein, in STSA-1 and CT1258 canine cancer cell lines. The results showed that all vaccinia virus strains were able to effectively infect, replicate in, and lyse the tested canine tumor cells. The virus infection led to efficient expression of FP635 under cell culture conditions. In addition, we showed, for the first time, that vaccinia virus strains can efficiently infect cAdMSCs at a lower MOI of 0.5. Interestingly, the virus replication in cAdMSCs was about 40–60-fold lower than in canine cancer cells at the same MOI (Figure 1; Figure 5). The reasons for the different virus replication in these cell types are currently under investigation.

The lysis of the virus-infected cAdMSCs seemed to be relatively delayed under the applied cell culture conditions. At a MOI of 0.5, only about 15% of the cells were lysed in the first two days of virus infection (Figure 7, LDH-release). Therefore, we decided to use an increased MOI for the animal studies. Interestingly, at a MOI of 10 h and 2 h after coincubation of C1-opt1 with cAdMSCs, we found about 96% living stem cells (Supplementary Table S1). For animal experiments, we chose C1-opt1 (Copenhagen strain), which was the most potent VACV strain among those tested when it comes to tumor cell lysis and replication speed in cAdMSCs.

The oncolytic effects of C1-opt1-loaded cAdMSCs and the C1-opt1 alone were tested in mice with STSA-1 tumor xenografts. The results demonstrated that both C1-opt1 alone and when loaded into the stem cells achieved a significant inhibition of tumor growth in the STSA-1 canine xenograft models. In the both cases, the STSA-1 tumor xenografts demonstrated a typical three-phase growth curve, and the tumor regression was observed as early as 14 days after virus injection, as described in previous studies [19,22]. However, combinational treatment resulted in slightly more efficient tumor regression compared to the group treated with C1-opt1 alone, in the time between 13 and 21 days post virus infection (Figure 8). The increased therapeutic efficacy of the C1-opt1 loaded cAdMSCs in the STSA-1 tumor xenografts might be a result of an enhanced virus delivery and/or protection of the virus by cAdMSCs.

The analysis of virus distribution in STSA-1 tumor-bearing nude mice at 24 dpvi revealed that C1-opt1 preferentially replicated in cancerous tissue (Table 2, Figure 9B,C). We also found a few virus particles in the lungs of most mice injected with C1-opt1/cAdMSCs but not with C1-opt1 alone (Table 2). One possible explanation for these results may be that a part of the C1-opt1/cAdMSCs was trapped in the lungs [23,24] and persisted in this organ for a short time after injection. The later presence of C1-opt1 in lungs might also be an indication for metastases formation in this organ. Moreover, it is known that vaccinia virus strains show a preferential colonization of metastases [25].

Generally, the C1-opt1 virus seems to be highly tumor-specific, since C1-opt1 was mainly detected in the tumor tissue of all treated animals (Table 2). Concerning biodistribution, the new used vaccinia virus Copenhagen strain C1-opt1 seems to be more tumor-specific compared to other vaccinia virus strains like GLV-1h68 [19], LIVP 1.1.1 [19], LIVP 6.1.1 [22], and GLV-5b451 [26], which were already successfully tested in STSA-1 xenografted mice.

Finally, the application of cAdMSCs in mice was not toxic and led to a moderate effect on the body weight of the injected animals (Figure 9B). Therefore, cAdMSCs could be useful as a “Trojan horse” for the delivery of oncolytic VACVs in vivo.

However, the therapeutic efficacy of combination of cAdMSCs and the C1-opt1 vaccinia virus strain should be confirmed in clinical trials with canine cancer patients.

## Figures and Tables

**Table 1:** Used FACS Antibodies.

Antibody/Fluorochrome	Dilution	Vendor/Cat. No./Clone
CD45 mouse IgG2a	1:100	Antikoerper-online.de, ABIN2664726, UCHL1
MHC II mouse IgG1	1:200	Bio-Rad, MCA1085GA, CVS20
CD44 rat IgG2b	1:400	BD Biosciences, 553131, IM7
CD90 rat IgG2b	1:50	Bio-Rad, MCA1036G, YKIX337.217
Anti-mouse polyclonal PE	1:200	Antikoerper-online.de, ABIN376443
Anti-rat polyclonal APC	1:600	BD Biosciences, 551019
Mouse IgG1 Isotype Control	1:200	Thermo Fisher Scientific, 02-6100
Mouse IgG2a Isotype Control	1:200	Thermo Fisher Scientific, 02-6200
Rat IgG2b Isotype Control	1:800	Thermo Fisher Scientific, 02-9288

**Table 2:** Virus distribution in STSA-1 tumor-bearing nude mice at 24 days post virus infection (dpvi).

PFU/per Gram (g) of Organ	STSA-1 Xenografts Treated with C1-opt1 Alone			STSA-1 Xenografts Treated with cAdMSCs/C1-opt1		
Mouse No	78	88	89	80	83	86
Tumor	4.80 × 10^4^	1.54 × 10^5^	5.78 × 10^5^	2.50 × 10^6^	3.83 × 10^5^	2.37 × 10^6^
Lung	n.d.	n.d.	n.d.	3.45 × 10^2^	3.45 × 10^2^	n.d
Liver	n.d.	n.d.	n.d.	n.d.	n.d.	n.d
Spleen	n.d.	n.d.	n.d.	n.d.	n.d.	n.d
Kidney	n.d.	n.d.	n.d.	n.d.	n.d.	n.d
Serum	n.d.	n.d.	n.d.	n.d.	n.d.	n.d

Tumor-bearing mice were injected with 2.5 × 10^6^ PFU of C1-opt1 alone or with a combination of 2.5 × 10^6^ PFU C1-opt1/2.5 × 10^5^ cAdMSCs. Mice were sacrificed at 24 dpvi. The data were determined by standard plaque assays on CV-1 cells using aliquots of the homogenized organs and were displayed as mean PFU/organ or tissue. For each organ, one aliquot of 50 µL was measured in duplicates. n.d.: Not detected (detection LIMIT < 10 pfu/organ).

## Supplementary Materials

The following are available online at https://www.mdpi.com/1999-4915/12/7/750/s1. Table S1: Cell viability of cAdMSCs alone or in combination C1-opt1/cAdMSCs. Supplementary Figure S1: Semi-quantitative WB analysis of the time-dependent expression of virus mediated FP635 in vaccinia virus infected canine cancer CT1258 cells (A) or in canine soft tissue sarcoma STSA-1 cells (B).

## References

[1] Patil S.S., Gentschev I., Nolte I., Ogilvie G., Szalay A.A. (2012). Oncolytic virotherapy in veterinary medicine: Current status and future prospects for canine patients. J. Transl. Med..

[2] Gentschev I., Patil S.S., Petrov I., Cappello J., Adelfinger M., Szalay A.A. (2014). Oncolytic virotherapy of canine and feline cancer. Viruses.

[3] MacNeill A.L. (2015). On the potential of oncolytic virotherapy for the treatment of canine cancers. Oncolytic Virother..

[4] Sanchez D., Cesarman-Maus G., Amador-Molina A., Lizano M. (2018). Oncolytic viruses for canine cancer treatment. Cancers (Basel).

[5] Ong H.T., Hasegawa K., Dietz A.B., Russell S.J., Peng K.W. (2007). Evaluation of t cells as carriers for systemic measles virotherapy in the presence of antiviral antibodies. Gene Ther..

[6] Komarova S., Kawakami Y., Stoff-Khalili M.A., Curiel D.T., Pereboeva L. (2006). Mesenchymal progenitor cells as cellular vehicles for delivery of oncolytic adenoviruses. Mol. Cancer Ther..

[7] Moreno R., Fajardo C.A., Farrera-Sal M., Perise-Barrios A.J., Morales-Molina A., Al-Zaher A.A., Garcia-Castro J., Alemany R. (2019). Enhanced antitumor efficacy of oncolytic adenovirus-loaded menstrual blood-derived mesenchymal stem cells in combination with peripheral blood mononuclear cells. Mol. Cancer Ther..

[8] Cejalvo T., Perise-Barrios A.J., Del Portillo I., Laborda E., Rodriguez-Milla M.A., Cubillo I., Vazquez F., Sardon D., Ramirez M., Alemany R. (2018). Remission of spontaneous canine tumors after systemic cellular viroimmunotherapy. Cancer Res..

[9] Power A.T., Wang J., Falls T.J., Paterson J.M., Parato K.A., Lichty B.D., Stojdl D.F., Forsyth P.A., Atkins H., Bell J.C. (2007). Carrier cell-based delivery of an oncolytic virus circumvents antiviral immunity. Mol. Ther..

[10] Greish K., Frandsen J., Scharff S., Gustafson J., Cappello J., Li D., O’Malley B.W., Ghandehari H. (2010). Silk-elastinlike protein polymers improve the efficacy of adenovirus thymidine kinase enzyme prodrug therapy of head and neck tumors. J. Gene Med..

[11] Fujiwara S., Nawa A., Luo C., Kamakura M., Goshima F., Kondo C., Kiyono T., Kikkawa F., Nishiyama Y. (2011). Carrier cell-based delivery of replication-competent hsv-1 mutants enhances antitumor effect for ovarian cancer. Cancer Gene Ther..

[12] Ikeda K., Ichikawa T., Wakimoto H., Silver J.S., Deisboeck T.S., Finkelstein D., Harsh G.R.t., Louis D.N., Bartus R.T., Hochberg F.H. (1999). Oncolytic virus therapy of multiple tumors in the brain requires suppression of innate and elicited antiviral responses. Nat. Med..

[13] Fisher K.D., Seymour L.W. (2010). Hpma copolymers for masking and retargeting of therapeutic viruses. Adv. Drug Deliv. Rev..

[14] Russell K.A., Chow N.H., Dukoff D., Gibson T.W., LaMarre J., Betts D.H., Koch T.G. (2016). Characterization and immunomodulatory effects of canine adipose tissue- and bone marrow-derived mesenchymal stromal cells. PLoS ONE.

[15] Reich C.M., Raabe O., Wenisch S., Bridger P.S., Kramer M., Arnhold S. (2012). Isolation, culture and chondrogenic differentiation of canine adipose tissue- and bone marrow-derived mesenchymal stem cells--a comparative study. Vet. Res. Commun..

[16] Fritz V., Jorgensen C. (2008). Mesenchymal stem cells: An emerging tool for cancer targeting and therapy. Curr. Stem Cell Res. Ther..

[17] Scioli M.G., Storti G., D’Amico F., Gentile P., Kim B.S., Cervelli V., Orlandi A. (2019). Adipose-derived stem cells in cancer progression: New perspectives and opportunities. Int. J. Mol. Sci..

[18] Draganov D.D., Santidrian A.F., Minev I., Nguyen D., Kilinc M.O., Petrov I., Vyalkova A., Lander E., Berman M., Minev B. (2019). Delivery of oncolytic vaccinia virus by matched allogeneic stem cells overcomes critical innate and adaptive immune barriers. J. Transl. Med..

[19] Gentschev I., Adelfinger M., Josupeit R., Rudolph S., Ehrig K., Donat U., Weibel S., Chen N.G., Yu Y.A., Zhang Q. (2012). Preclinical evaluation of oncolytic vaccinia virus for therapy of canine soft tissue sarcoma. PLoS ONE.

[20] Fork M.A., Murua Escobar H., Soller J.T., Sterenczak K.A., Willenbrock S., Winkler S., Dorsch M., Reimann-Berg N., Hedrich H.J., Bullerdiek J. (2008). Establishing an in vivo model of canine prostate carcinoma using the new cell line ct1258. BMC Cancer.

[21] Muller M., Raabe O., Addicks K., Wenisch S., Arnhold S. (2011). Effects of non-steroidal anti-inflammatory drugs on proliferation, differentiation and migration in equine mesenchymal stem cells. Cell Biol. Int..

[22] Gentschev I., Patil S.S., Adelfinger M., Weibel S., Geissinger U., Frentzen A., Chen N.G., Yu Y.A., Zhang Q., Ogilvie G. (2013). Characterization and evaluation of a new oncolytic vaccinia virus strain livp6.1.1 for canine cancer therapy. Bioengineered.

[23] Fischer U.M., Harting M.T., Jimenez F., Monzon-Posadas W.O., Xue H., Savitz S.I., Laine G.A., Cox C.S. (2009). Pulmonary passage is a major obstacle for intravenous stem cell delivery: The pulmonary first-pass effect. Stem Cells Dev..

[24] Schrepfer S., Deuse T., Reichenspurner H., Fischbein M.P., Robbins R.C., Pelletier M.P. (2007). Stem cell transplantation: The lung barrier. Transpl. Proc..

[25] Donat U., Weibel S., Hess M., Stritzker J., Hartl B., Sturm J.B., Chen N.G., Gentschev I., Szalay A.A. (2012). Preferential colonization of metastases by oncolytic vaccinia virus strain glv-1h68 in a human pc-3 prostate cancer model in nude mice. PLoS ONE.

[26] Adelfinger M., Bessler S., Frentzen A., Cecil A., Langbein-Laugwitz J., Gentschev I., Szalay A.A. (2015). Preclinical testing oncolytic vaccinia virus strain glv-5b451 expressing an anti-vegf single-chain antibody for canine cancer therapy. Viruses.

