# Supplementary material for: Canine Adipose-Derived Mesenchymal Stem Cells (cAdMSCs) as a “Trojan Horse” in Vaccinia Virus Mediated Oncolytic Therapy against Canine Soft Tissue Sarcomas
Source: Viruses. 2020 Jul 12;12(7):750. doi: 10.3390/v12070750 (PMC7411685; doi:10.3390/v12070750)
Supplement: Supplementary file 1 [file viruses-12-00750-s001.zip › viruses-815602 supplementary/Figure S1.pdf]

A

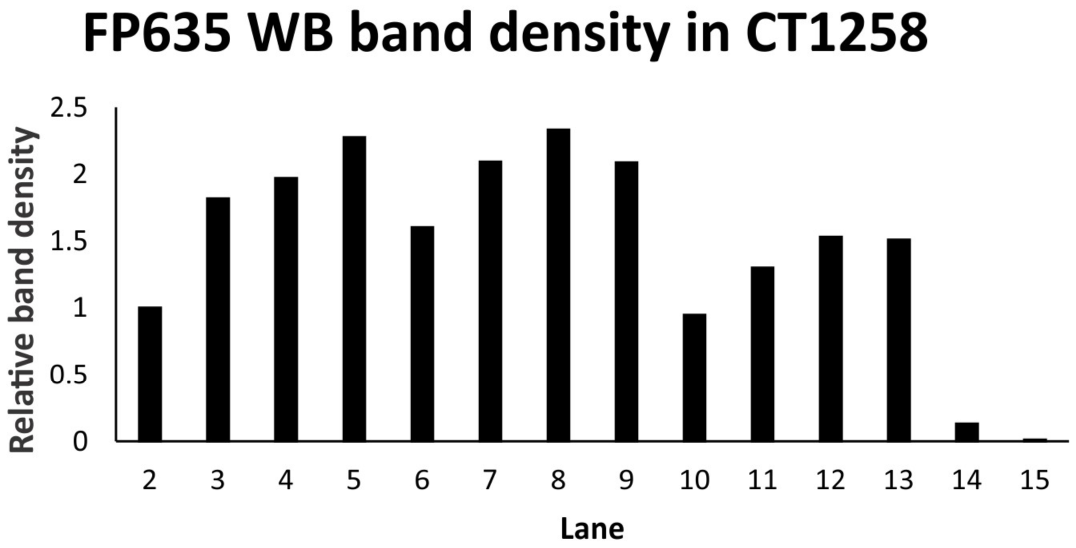

B

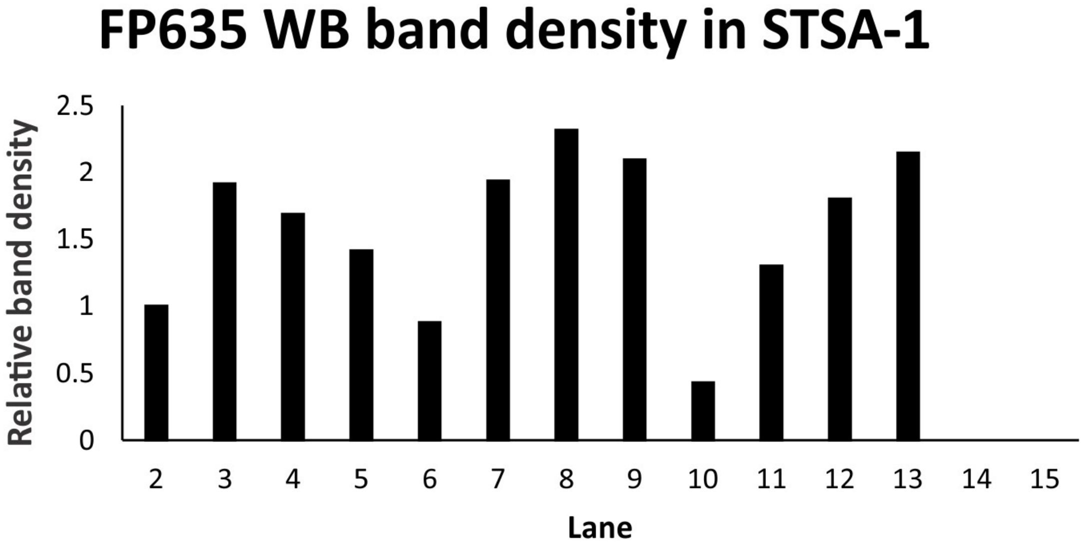

Supplementary Figure 1. Semi-quantitative western blot analysis of the time-dependent expression of virus mediated FP635 in vaccinia virus infected canine cancer CT1258 cells (A) or in canine soft tissue sarcoma STSA-1 cells (B). Lanes 2-5: L3-opt1 infected cells; lanes 6-9: C1-opt1 infected cells; lanes 10-13: W1-opt1 infected cells; lanes 14-15: non-infected CT1258 or STSA-1 cells (negative controls). Western blot was analysed using ImageJ Gel Analysis tool, band densities were quantified and normalized to band density in lane 2 (L3-Opt1 infected cells, 24 hpi).
