# Supplementary material for: Canine Adipose-Derived Mesenchymal Stem Cells (cAdMSCs) as a “Trojan Horse” in Vaccinia Virus Mediated Oncolytic Therapy against Canine Soft Tissue Sarcomas
Source: Viruses. 2020 Jul 12;12(7):750. doi: 10.3390/v12070750 (PMC7411685; doi:10.3390/v12070750)
Supplement: Supplementary file 1 [file viruses-12-00750-s001.zip › viruses-815602 supplementary/Table S1.pdf]

Supplementary Table S1: Cell viability of cAdMSCs alone or in combination C1-opt1/cAdMSCs

**Living cAdMSCs in percentages (%)**	**cAdMSCs alone**	**Combination C1-opt1/ cAdMSCs**
Time point = 0h	99%	n.t
Time point = 2h	n.t	96%

cAdMSCs alone or combination C1-opt1/cAdMSCs (MOI =10) were incubated in DMEM medium, supplemented with 1% fetal calf serum for 2 hours at 37°C. The probes were stained with 0.40% Trypan Blue Solution (Bio Rad,Germany, cat. No. 1450013) and the cell viability was analyzed with TC20™ cell couter (Bio Rad, Munich, Germany). n.t.: not tested
